# The impact of manipulating personal standards on eating attitudes and behaviour

**DOI:** 10.1016/j.brat.2005.08.009

**Published:** 2006-06

**Authors:** Roz Shafran, Michelle Lee, Elizabeth Payne, Christopher G. Fairburn

**Affiliations:** Oxford University Department of Psychiatry, Warneford Hospital, Oxford OX3 7JX, UK

**Keywords:** Eating disorders, Theory, Experiment, Perfectionism

## Abstract

The relationship between perfectionism and eating disorders is well established and is of theoretical interest. This study used an experimental design to test the hypothesis that manipulating personal standards, a central feature of perfectionism, would influence eating attitudes and behaviour. Forty-one healthy women were randomly assigned either to a high personal standards condition (n=18) or to a low personal standards condition for 24 h (n=23). Measures of personal standards, perfectionism, and eating attitudes and behaviour were taken before and after the experimental manipulation. The manipulation was successful. After the manipulation, participants in the high personal standards condition ate fewer high calorie foods, made more attempts to restrict the overall amount of food eaten, and had significantly more regret after eating than those in the low personal standards condition. Other variables remained unchanged. It is concluded that experimental analyses can be of value in elucidating causal connections between perfectionism and eating attitudes and behaviour.

## Introduction

The association between perfectionism and eating disorder psychopathology is of interest for several reasons. Perfectionism co-occurs with eating disorders (Bulik et al., 2003; Halmi et al., 2000), the construct of perfectionism is considered to be characteristic of eating disorders, in particular anorexia nervosa (Garner, Olmsted, & Polivy, 1983; Vitousek & Manke, 1994) and it has an important role in recent theories of the maintenance of eating disorders (Fairburn, Cooper, & Shafran, 2003). Perfectionism has been identified as a specific risk factor for the development of bulimia nervosa and anorexia nervosa (Fairburn, Cooper, Doll, & Welch, 1999; Lilenfield et al., 1998) and bulimic symptoms (Joiner, Heatherton, Rudd, & Schmidt, 1997). Childhood obsessive-compulsive personality traits including perfectionism, have also been shown to be predictive of the development of eating disorders in adult life (Anderluh, Tchanturia, Rabe–Hesketh, & Treasure, 2003).

Another reason for interest in the relationship between perfectionism and eating attitudes and behaviour concerns recent developments in theories concerning the nature of perfectionism. In response to the lack of treatment advances deriving from multidimensional analyses of perfectionism, the construct of “clinical perfectionism” was proposed (Shafran, Cooper, & Fairburn, 2002). Clinical perfectionism is a notion designed to capture a specific type of perfectionism that poses a clinical problem. It is defined as the over-evaluation of the striving for, and achievement of, personally demanding standards, despite adverse consequences. According to this account, eating disorders and clinical perfectionism have a particular connection whereby for some patients, the eating disorder is an expression of clinical perfectionism in the domain of eating, shape and weight, and their control. Despite some debate as to the utility of the construct of clinical perfectionism (Hewitt, Flett, Besser, Sherry, & McGee, 2003; Shafran, Cooper, & Fairburn, 2003), a qualitative analysis of clinical perfectionism and a case series of its treatment provide support for the construct (Glover, Riley, Shafran, Brown, & Fairburn, 2004; Riley & Shafran, 2005).

A key prediction deriving from the analysis of clinical perfectionism is that manipulating clinical perfectionism should have an impact on eating attitudes and behaviour in some cases. Although it is difficult to manipulate clinical perfectionism, it is possible to manipulate high standards for performance in an analogue paradigm. It was decided to manipulate high standards for performance rather than other aspects of clinical perfectionism (for example, the extent to which self-worth is based on achievement or self-criticism) for two reasons. First, the literature indicates that people with eating disorders have higher personal standards than psychiatric controls (Cockell et al., 2002). Second, pursuit of personally demanding/high standards is necessary (although not sufficient) for all approaches to dysfunctional perfectionism regardless of whether the theoretical model is multidimensional (Frost et al., 1991; Hewitt & Flett, 1991) or the more focused clinical construct (Shafran et al., 2002).

The aim of this analogue study was therefore to experimentally manipulate personal standards, and determine the effects on eating attitudes and behaviour. It was hypothesised that participants who were asked to attempt to reach high standards and stringently adhere to them would show more dysfunctional eating attitudes and behaviour compared to participants who were assigned to have low personal standards.

## Method

### Participants

**Fig. 1:**
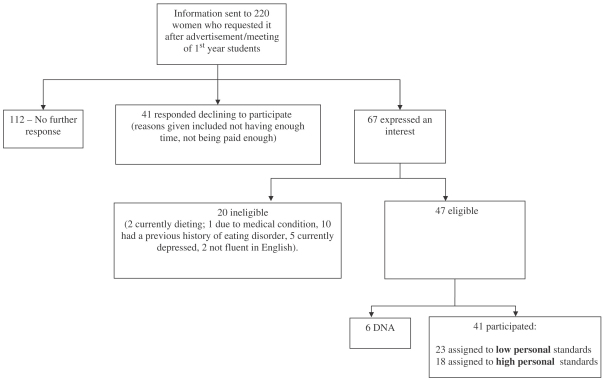
Recruitment of participants.

A total of 220 women were sent information after either responding to an advertisement for volunteers to participate in research on eating behaviour and perfectionism or indicating an interest in participating in clinical research at a first year meeting of university students. A flow chart outlining recruitment is provided (Fig. 1). Of the 47 who agreed to participate and were eligible, 41 kept their appointment. The mean age of this final sample was 22.6 years (SD=3.97, range 19–42). Exclusion criteria included current or past history of an eating disorder, current dieting (<1500 kcal per day), medical conditions affecting appetite or currently significantly depressed (total score on the Beck Depression Inventory-II above 30, or 2 or above on the item assessing suicidal tendencies).

### Questionnaires/assessments

#### Eating Disorder Examination—Questionnaire version (EDE-Q; Fairburn & Beglin, 1994)

This self-report questionnaire is based on the EDE (Cooper & Fairburn, 1987) and focuses on the participant's state over the preceding 28 days. It uses a 7-point forced-choice rating scheme. The questionnaire has good reliability and validity (e.g., Mond, Hay, Rodgers, Owen, & Beumont, 2004) and at the end, participants are asked to report their weight and height.

#### Beck Depression Inventory II (BDI-II; Beck, Steer, & Brown, 1996)

This is a 21-item self-report instrument for measuring the severity of depression. The measure assesses symptoms corresponding to the diagnostic criteria for depressive disorder specified in DSM-IV (American Psychiatric Association, 1994). Each item is scored from 0 to 3, and the total score is the sum of each item. Its reliability and validity are well established (Beck et al., 1996).

#### Clinical Perfectionism Questionnaire (CPQ: Fairburn et al., 2003)

This 12-item measure assesses the main features of clinical perfectionism over the previous month. The items include assessing the extent to which an individual's self-worth is based on their ability to achieve high standards, whether they have missed out on any activities due to their attempts to reach their goals, and the extent to which they have felt a failure. Items are rated on a 4-point scale (1—not at all to 4—all the time). It has good internal consistency (α=.78) and discriminant validity (Fairburn, Cooper, Lee, & Shafran, in preparation).

#### Multidimensional Perfectionism Scale (MPS; Hewitt & Flett, 1991)

This is a 45-item measure of perfectionism in general. It assesses self-oriented perfectionism (MPS-self, i.e., self-imposed standards), socially prescribed perfectionism (MPS-social; standards the respondent perceives others set him/her) and other-oriented perfectionism (MPS-other; standards for other people). Each item is rated on a 7-point Likert scale ranging from 1 (disagree) to 7 (agree). The MPS has acceptable test–retest reliability and construct validity of .80–.86 (Hewitt & Flett, 1991).

#### Visual Analogue Scales (VAS)

Two VAS were constructed for the purposes of this study. Both asked about the ‘previous 24 h’ and were administered immediately before, and 24 h after, the experimental manipulation. For both scales, participants were asked to place a cross on a 10 cm horizontal scale to indicate their level of agreement with each item. The scales ranged from 0 (“not at all”) to 100 (“totally”). The first VAS comprised nine key items that assessed clinical perfectionism (referred to here as the CP-VAS; e.g., ‘To what extent have you judged yourself on the basis of your ability to reach high standards?’; ‘How afraid have you been that you might not reach your standards?’, ‘How much have you been determinedly pursuing high (personally demanding) standards?’). One item within this scale (‘To what extent have you been pursuing low (personally undemanding) standards?’) was reverse-scored. The second scale assessed eating behaviour and comprised four items that assessed attempted and actual dietary restraint (in terms of overall amounts of food eaten) and attempted and actual exclusion (the avoidance of specific desired foods) based on specific items within the EDE (Cooper & Fairburn, 1987) (i.e., ‘How much of the time have you *actually* been excluding any foods which you like?’ and ‘How much of the time have you been *trying* to cut back or restrict the overall amount of food that you eat (whether or not you have actually succeeded)?)’. VAS have been shown to have good reliability and validity in a range of settings (McCormack, Horne, & Sheather, 1988).

#### Guilt, regret and over-evaluation of control

Based on items within the EDE, participants were asked to rate their degree of regret and guilt after eating, and the importance of controlling their eating over the previous 24 h. Responses were rated by the assessor using a scale similar to that used in the EDE (from 0 to 6).

#### Food consumption

Participants were asked to describe exactly what they had eaten over the 24 h period immediately prior to, and 24 h, after the experimental manipulation. Foods high and low in calories/fat were subsequently categorised by an experimenter (EP) (blind to each participant's group allocation). Examples of high calorie/fat foods included fast foods, e.g., chips and pizza, sweet foods such as chocolate and biscuits, and dairy products such as cream and cheese. Examples of low calorie/fat foods included fruit and vegetables, salad (without dressing) and reduced calorie/fat alternatives (such as ‘no sugar’ chocolate). The total number of high fat/calorie food items and low fat/calorie food items eaten in the 24 h period prior to the experiment was summed. This was also done for the 24 h period after the experimental manipulation.

#### Manipulation checks

Two manipulation checks were made. First, the degree to which participants responded to the item on the CP-VAS ‘How much have you been determinedly pursuing high (personally demanding) standards?’ in the 24 h before and after the experimental manipulation. Second, participants rated the extent to which they had been following their contract (using a VAS from 0 “not at all” to 100 “totally”).

### Procedure

Participants completed the EDE-Q, BDI-II, CPQ and MPS on one occasion, within the 24 h prior to attending the first session. At the first session, participants were asked to describe their typical eating pattern over the previous month, in the same way as asked in the EDE, and to go through what they had eaten in the previous 24 h, in terms of actual foods and amounts. Participants completed the CP-VAS and the four-item eating behaviour VAS. Guilt after eating, regret after eating and importance of control over eating were assessed for the previous 24 h (prior to the experimental manipulation) and rated by an assessor (ML). Participants were then introduced to a second experimenter (EP) and were randomly assigned to either the low personal standards condition or the high personal standards condition. The second experimenter asked the participant to sign and complete one of the following two contracts (based on Lopatka & Rachman, 1995) depending on their group assignment. The relevant contract was discussed and specific examples of behaviour using high or low standards were elicited by the experimenter. For example, in the low personal standards condition, participants might be asked to take it easy at work (take regular breaks, put off non urgent tasks, leave on time or early and surf the internet, etc.), whereas in the high personal standards condition participants might be asked to work very hard (get in early, make a “to-do” list and make sure everything was ‘ticked off’, take minimal breaks, answer emails and calls promptly, stay later than normal, etc.). Clauses relating to the type or amount of food eaten during this period were not included.

#### High personal standards contract

This is a contract made between _________(participant) and ___________ (researcher) on _____ (date). I ___________ (participant) confirm that for the next 24 h, EVERYTHING that I do will be done to the HIGHEST POSSIBLE STANDARD. This will include everything that I think and say and do, as discussed with the researcher, named above. I understand what I am required to do and agree to do this consistently throughout the next 24-h period. In particular I agree to (up to 10 items were specified):

(1). …(Example: Be either early or at least on time for EVERYTHING);
(2). …(Example: Drive perfectly (e.g. always using signals) and park perfectly. If when parking in the street you are slightly wonky, do it again until you are completely parallel with the pavement.);
(3). …(Example: Be the perfect hostess to you mother, who is staying with you tonight).
  ….
(10). …

#### Low personal standards contract

This was the same as above except that the word ‘HIGHEST’ was replaced with ‘MINIMAL’.

Participants returned for another assessment 24 h after the experimental manipulation. The assessor was blind to the experimental condition of the participants. Participants again completed the CP-VAS and eating attitudes/behaviour, and described what they had eaten over the previous 24 h. The experimenter did not look at the completed CP-VAS in order to maintain blindness of group allocation. The assessment questions relating to regret after eating, guilt after eating and importance of control over eating were completed. After this, the condition to which the participant had been assigned was revealed to the assessor who went through the contract clause by clause to determine the participants’ degree of adherence, and finally the assessor asked participants to complete the second manipulation check VAS (the extent to which the contract had been followed from 0 to 100). The study was approved by the local psychiatric research ethics committee, and written consent was obtained from all participants before taking part.

## Results

### Participant characteristics

The mean age, BMI and questionnaire scores for participants in each condition are shown in Table 1. The participants in the two conditions did not differ significantly from each other on any of these measures prior to the experimental manipulation (all p>.05).

### Manipulation check

#### Check 1: VAS item from the CP-VAS to assess extent to which participant had been striving to reach high standards.

In the 24 h post-manipulation, paired *t*-tests revealed a significant decrease in striving to reach high personal standards after the manipulation for those in the low personal standards condition (M=53.13 (SD=20.91) vs. M=11.52 (SD=17.24); t(22)=6.25, p<.001) and a significant increase in striving to reach high personal standards in those in the high personal standards condition (M=53.72 (SD=28.40) vs. M=84.77 (SD=12.51); t(17)=4.15, p<.005). An independent *t*-test revealed a significant difference between the two groups post manipulation (t(39)=15.16, p<.001).

#### Check 2: VAS item to assess adherence to the contract.

There was no difference in the self-report ratings of the two groups concerning the extent to which they adhered to the contract (M=80.0 in the low personal standards condition vs. M=78.7 in the high personal standards condition; SD=15.1 and 8.8, respectively; t(39)=.33, p>.05).

### Impact of the manipulation on specific aspects of clinical perfectionism

To assess the impact of the manipulation on the specific aspects of clinical perfectionism, group differences on individual items within the CP-VAS were examined prior to and after the manipulation. The relevant means (and standard deviations) are presented in Table 2. Prior to the manipulation, the two groups did not differ from each other on any item within the CP-VAS. After the experimental manipulation, compared to those in the low personal standards condition, those in the high personal standards condition reported increased striving (pushing hard to achieve standards), fear of failure, over-evaluation in terms of achieving standards, performance checking behaviour, missing out on things because of trying to achieve their standards, dichotomous thinking, reduced pursuit of undemanding standards and a reduction in the noticing of success (all *p*'s<.005, except item 8 (dichotomous thinking), where p<.05). These findings indicate that the manipulation had an impact on all the key components of clinical perfectionism.

### Impact of the experimental manipulation on eating attitudes and behaviour

The mean scores on the measures of eating attitudes and behaviour, prior to and after the experimental manipulation, are reported in Table 3. Exploratory data analysis indicated that the variables of attempted and actual restraint, attempted and actual exclusion, regret, guilt and importance of control over eating were not normally distributed (due to significant positive skews), therefore, where appropriate, comparisons of groups pre- and post-manipulation were conducted with non-parametric Mann–Whitney tests. Prior to the manipulation, the two groups did not differ from each other on any measure as indicated in Table 3. After the manipulation, compared to those in the low personal standards condition, those in the high personal standards condition ate significantly fewer high calorie foods, reported significantly more attempts to restrict the overall amount of food eaten, and reported significantly more regret after eating. No significant differences were found between the groups on actual or attempted dietary exclusion, actual restriction, number of low calorie foods consumed, guilt after eating and importance of control over eating. Of interest, after the manipulation 13/41 (32%) of the participants reported their actual restriction had changed by 20 or more points on the eating behaviour VAS and 21/41 (51%) reported their attempts to restrict changed by 20 or more points on this scale. Similar proportions were reported for actual dietary exclusion (9/41, i.e., 22%) and attempted dietary exclusion (20/41, i.e., 49%).

### Relationship between clinical perfectionism, multidimensional perfectionism and eating attitudes/behaviour and depression

The relationship between eating psychopathology, depression, clinical perfectionism and multidimensional perfectionism was assessed and correlation coefficients are presented in Table 4. There were significant correlations between the multidimensional perfectionism subscales and the clinical perfectionism questionnaire (r=.42–.66; p<.01) but not between these measures and eating disorder attitudes/behaviour (all *p*'s>.05) with the exception of MPS-social scores and EDE-Q Eating Concern scores (r=.36, p<.05). Level of depression was significantly correlated with EDE-Q Eating Concern scores, Shape Concern scores and Weight Concern scores, as well as MPS-social scores (all *p*'s<.01). The significant correlations between the CPS and MPS remained after controlling for level of depression (r=.37–.62, all *p*'s<.05).

## Discussion

The main aim of this study was to test the hypothesis that participants who actively attempt to have high personal standards and stringently adhere to them will show more dysfunctional eating attitudes and behaviour than those who are not attempting to adhere to high personal standards. The experimental manipulation was successful and made such a test possible. Those in the low personal standards condition behaved with low standards and those who agreed to act according to extremely high personal standards also met their obligations. After the manipulation, those in the high personal standards condition ate fewer high calorie foods than those in the low personal standards condition, they made significantly more attempts to restrict the overall amount of food eaten, and had significantly more regret after eating. The manipulation did not significantly influence feelings of wrongdoing after eating (i.e., guilt after eating), attempted or actual exclusion or actual restriction of the overall amount of food eaten or the number of low fat/calorie foods consumed. Unlike previous research (Bulik et al., 2003; Flett & Hewitt, 2002), no association was found between the level of dysfunctional eating attitudes/behaviour and the measures of perfectionism. It is unlikely that this is solely due to the non-clinical nature of the sample since such associations have been found previously but the findings may also be in part due to the particular measures used in this study. Given the debate regarding the relationship between multidimensional and clinical perfectionism (e.g., Hewitt et al., 2003) the significant correlations between two of the subscales of the MPS and the clinical perfectionism questionnaire is of interest and warrants further exploration.

The present findings are consistent with the model of clinical perfectionism although they cannot be considered as a direct test for two reasons. First, clinical perfectionism was not manipulated per se. Rather, the study manipulated one aspect of clinical perfectionism, i.e., high personal standards. Given the difficulty in manipulating clinical perfectionism per se, it was necessary to choose one aspect of the construct. Personal standards were chosen as they are universally accepted to be integral to the construct of perfectionism. It would have been possible and desirable to manipulate other aspects of clinical perfectionism, in particular the self-criticism that has been found to be necessary to combine with the personal standards to produce the type of perfectionism that poses a clinical problem (Dunkley, Blankstein, Masheb, & Grilo, in press). Future studies manipulating and the combination of personal standards and self-criticism would provide a directly relevant test of the model of clinical perfectionism. Having said that, the data from the clinical perfectionism VAS indicated that although the manipulation was focused on personal standards, the other variables relevant to clinical perfectionism also changed. Importantly for the model of clinical perfectionism, there was a difference between the groups on the item assessing the extent to which the self-worth was dependent on achieving high standards. The manipulation therefore indirectly altered what is considered to be the ‘core psychopathology’ of clinical perfectionism. There was also a significant difference between groups on each of the variables considered to assess the construct of clinical perfectionism (Table 2). As such, it is not unreasonable to extrapolate the findings from the experimental manipulation of high standards to the experimental manipulation of clinical perfectionism more broadly.

A second reason that the findings are not a direct test of the model is that the theory predicts that eating disorder psychopathology can *in some cases* be viewed as, in part, an expression of clinical perfectionism in the domain of eating, shape, weight or their control. This prediction was tested using an analogue sample that did not investigate individual differences with the exception of the finding that approximately one-third of participants reported a change (more than 20 points out of 100 on the visual analogue scale) in their actual dietary restriction after the manipulation and one-half reported a change in their attitudes towards dietary restriction. Why there is such a connection between perfectionism and eating in some people but not others is a question worthy of further exploration.

This study had a number of other limitations. Although it was designed to manipulate personal standards in general and determine the resulting effect on eating attitudes and behaviour, it was inescapable that participants would realise that eating was a variable of interest and some of them had responded to an advert stating this explicitly. Therefore there is a possibility that these findings may be due to experimenter demand. A study that embedded eating behaviour among other variables would help overcome this limitation. In addition, it would have been desirable to include a debriefing interview asking participants whether they had assumed that what was being included on the contract was their eating behaviour regardless of whether or not it had actually been mentioned during the study. Comparing whether eating behaviour was specifically affected by the manipulation relative to other behaviours, e.g., drinking of alcohol, exercising, could also have been included to help rule out the possibility that the results were only due to participants having insight into the nature of the study. On the other hand, attempts were made to reduce experimenter demand by having a second assessor who had not been involved in the experimental manipulation. Furthermore, if the results were due solely to experimenter demand, it is not unreasonable to assume that this would have been reflected in changes in reported eating across all the variables, and not just the particular ones reported above. Another limitation is that this study was conducted on healthy women only and not those with eating disorders. This was for two reasons. First, ethically it would not have been acceptable to potentially exacerbate an eating disorder in a clinical sample. Second, we wished to be able to both increase and decrease personal standards, and doing so might have proved more difficult in patients with eating disorders. The use of a healthy sample, however, limits the implications that can be drawn for clinical samples, and it remains to be tested whether the same effects would be observed in a clinical group with high levels of eating disorder psychopathology as opposed to mildly dysfunctional eating attitudes and behaviour. Finally, this study did not directly manipulate clinical perfectionism as such, but rather one aspect of it.

Despite the limitations, the strength of the study lies in the use of a highly focused, experimental method. Such experimental analyses move beyond descriptive data obtained by questionnaire or interviews, and provide a more specific test of hypotheses than is possible from treatment manipulations. It is suggested that further such analyses could play an important part in illuminating the nature of perfectionism, eating disorder psychopathology and the relationship between them.

## Figures and Tables

**Table 1:** Participant characteristics (means and standard deviations) pre-manipulation

	Highest possible standard (n=18)	Minimal possible standard (n=23)
Age	22.8 (4.6)	22.4 (3.2)
EDE-Q restraint	1.3 (1.1)	1.6 (1.1)
EDE-Q eating concern	1.0 (1.2)	.9 (1.2)
EDE-Q shape concern	2.5 (1.5)	2.4 (1.7)
EDE-Q weight concern	1.9 (1.4)	1.8 (1.3)
BDI-II	6.0 (5.6)	7.7 (7.2)
BMI	23.6 (4.4)	21.9 (3.3)
Clinical Perfectionism Questionnaire	25.0 (4.6)	23.5 (3.6)
MPS self	68.8 (16.9)	63.8 (13.3)
MPS other	58.3 (14.1)	59.5 (10.1)
MPS social	51.6 (15.5)	53.3 (12.5)

**Table 2:** Clinical perfectionism VAS scores (means and standard deviations) pre- and post-manipulation*

Aspect of clinical perfectionism	Pre-manipulation		Post-manipulation	
	Low standards	High standards	Low standards	High standards
Determinedly pursuing high standards	53.1 (20.9)	53.7 (28.4)	11.5 (17.2)	87.8 (12.5)
Pursuing low, undemanding standards	30.4 (23.4)	25.2 (26.2)	78.1 (15.1)	9.4 (16.8)
Pushing self hard to achieve standards	50.3 (25.6)	53.7 (21.2)	23.7 (25.2)	73.6 (21.8)
Fear of failure	36.6 (30.6)	45.4 (24.1)	22.9 (24.9)	70.4 (28.8)
Over-evaluation of achieving standards	37.1 (29.6)	51.7 (22.4)	17.7 (21.9)	75.6 (18.9)
Tendency to check actions	28.13 (26.7)	44.5 (28.9)	32.1 (33.4)	68.6 (25.3)
Missing out on things	16.0 (19.6)	26.0 (23.9)	11.3 (16.8)	48.0 (32.6)
Dichotomous thinking	31.4 (21.1)	22.9 (18.2)	26.5 (27.0)	49.4 (31.8)
Noticing successes	42.1 (26.0)	50.4 (23.2)	28.3 (23.5)	61.9 (24.0)

*A series of independent *t*-tests indicated that pre-manipulation, there were no group differences prior to the experimental manipulation on any variable (df=39, all *t*'s<1.9, all *p*'s>.05). Post-manipulation, there were group differences for all aspects of clinical perfectionism (all *p*'s<.001 except for dichotomous thinking where *p*<.05).

**Table 3:** Eating attitudes and behaviours pre- and post-manipulation (standard deviations in parentheses)*

Measure (previous 24 h)	Pre-manipulation		Post-manipulation	
	Low standards	High standards	Low standards	High standards
Number of high calorie foods eaten	2.2 (1.2)	1.8 (1.5)	2.5 (1.6)	1.5 (1.4)
Number of low calorie foods eaten	.8 (.6)	.6 (.7)	.7 (0.7)	.8 (0.7)
*Visual Analogue Scales*				
Actual exclusion	18.5 (26.0)	10.1 (18.7)	8.9 (14.3)	9.1 (15.4)
Attempted exclusion	26.6 (30.8)	28.4 (32.3)	22.9 (28.0)	17.9 (17.5)
Actual restriction	10.0 (18.8)	11.7 (19.9)	8.7 (15.0)	14.5 (20.5)
Attempted restriction	20.2 (24.5)	22.9 (30.5)	13.7 (25.0)	32.9 (25.4)
*Rating by (blind) assessor*				
Regret after eating	1.8 (1.6)	1.1 (1.7)	.8 (1.3)	2.0 (1.6)
Guilt after eating	.3 (.6)	.2 (.9)	.6 (1.6)	.3 (1.0)
Over-evaluation of control over eating	.8 (1.2)	.5 (1.2)	.7 (1.4)	1.2 (1.1)

*A series of Mann–Whitney and *t*-tests (where appropriate) indicated that pre-manipulation, there were no group differences prior to the experimental manipulation on any variable (df=39, all *p*'s>.05). Post-manipulation, there were group differences for attempted restraint (Z=1.54, p<.01), regret after eating (Z=2.31, p<.05) and number of high calorie foods eaten (t(39)=2.18, p<.05).

**Table 4:** Correlations between measures of perfectionism and eating attitudes and behaviour

	EDE restraint	EDE eating concern	EDE shape concern	EDE weight concern	MPS self	MPS other	MPS social	CPQ	CP-VAS (Session 1)
EDE eating concern	.36*	—							
EDE shape concern	.60**	.64**	—						
EDE weight concern	.56**	.70**	.86**	—					
MPS—self	.03	.15	.10	.12	—				
MPS—other	−.10	.26	.11	.11	.52**	—			
MPS—social	−.06	.36*	.20	.19	.54**	.53**	—		
CPS	.06	.11	.08	.18	.66**	.42**	.45**	—	.46**
CP-VAS (Session 1)	.33*	.22	.06	.06	.36*	.31	.41**	—	—
BDI	.18	.49**	.54**	.43**	.04	.24	.34**	.09	.18

*N*=41, *p<.05, **p<.01.

## References

[bib1] American Psychiatric Association (1994). Diagnostic and statistical manual of mental disorders.

[bib2] Anderluh M.B., Tchanturia K., Rabe-Hesketh S., Treasure J. (2003). Childhood obsessive-compulsive personality traits in adult women with eating disorders: Defining a broader eating disorder phenotype. American Journal of Psychiatry.

[bib3] Beck A.T., Steer R.A., Brown G. (1996). Beck depression inventory II.

[bib4] Bulik C.M., Tozzi F., Anderson C., Mazzeo S.E., Aggen S., Sullivan P.F. (2003). The relation between eating disorders and components of perfectionism. American Journal of Psychiatry.

[bib27] Cockell S.J., Hewitt P.L., Seal B., Sherry S., Goldner E.M., Flett G.L., Remick R.A. (2002). Trait and self-presentational dimensions of perfectionism among women with anorexia nervosa. Cognitive Therapy and Research.

[bib5] Cooper Z., Fairburn C.G.F. (1987). The eating disorders examination: A semi structured interview for the assessment of the specific psychopathology of eating disorders. International Journal of Eating Disorders.

[bib6] Dunkley, D. M., Blankstein, K. R., Masheb, R. M., Grilo, C. Personal standards and evaluative concerns dimensions of “clinical” perfectionism: A reply to Shafran et al. (2002, 2003) and Hewitt et al. (2003). *Behaviour Research and Therapy*, in press, doi:10.1016/j.brst.2004.12.004. 10.1016/j.brat.2004.12.004 16301015

[bib7] Fairburn C.G., Beglin S.J. (1994). Assessment of eating disorders: Interview or self-report questionnaire?. International Journal of Eating Disorders.

[bib8] Fairburn C.G., Cooper Z., Doll H.A., Welch S.L. (1999). Risk factors for anorexia nervosa: Three integrated case-control comparisons. Archives of General Psychiatry.

[bib9] Fairburn, C. G., Cooper, Z., Lee, M., Shafran, R. (in preparation). Psychometric properties of the Clinical Perfectionism Scale.

[bib10] Fairburn C.G., Cooper Z., Shafran R. (2003). Cognitive behaviour therapy for eating disorders: A “transdiagnostic” theory and treatment. Behaviour Research and Therapy.

[bib12] Flett G.L., Hewitt P.L. (2002). Perfectionism: Theory, research, and treatment.

[bib13] Garner D.M., Olmsted M.P., Polivy J. (1983). The eating disorder inventory.

[bib14] Glover, D., Riley, C., Shafran, Brown, G., Fairburn, C. G. (2004). CBT for clinical perfectionism: A case series. Poster presented at the *European Association for Behaviour and Cognitive Psychotherapy annual conference*, September 2004.

[bib15] Halmi K.A., Sunday S.R., Strober M., Kaplan A., Woodside D.B., Fichter M. (2000). Perfectionism in anorexia nervosa: Variation by clinical subtype, obsessionality, and pathological eating behavior. American Journal of Psychiatry.

[bib16] Hewitt P.L., Flett G.G., Besser A.B., Sherry S.B., McGee B. (2003). Perfectionism is multidimensional: A reply to Shafran, Cooper, and Fairburn. Behaviour Research and Therapy.

[bib17] Hewitt P.L., Flett G.L. (1991). Perfectionism in the self and social contexts: Conceptualization, assessment, and association with psychopathology. Journal of Personality and Social Psychology.

[bib18] Joiner T.E., Heatherton T.F., Rudd M.D., Schmidt N.B. (1997). Perfectionism, perceived weight status, and bulimic symptoms: Two studies testing a diathesis-stress model. Journal of Abnormal Psychology.

[bib19] Lopatka C., Rachman S. (1995). Perceived responsibility and compulsive checking: An experimental analysis. Behaviour Research and Therapy.

[bib20] Lilenfield L.R., Kaye W.H., Greeno C.G., Merikangas K.R., Plotnicov K., Pollice C. (1998). A controlled family study of anorexia nervosa and bulimia nervosa: Psychiatric disorders in first-degree relatives and effects of proband comorbidity. Archives of General Psychiatry.

[bib21] McCormack H.M., Horne D.J., Sheather S. (1988). Clinical applications of visual analogue scales: A critical review. Psychological Medicine.

[bib22] Mond J.M., Hay P.J., Rodgers B., Owen C., Beumont P.J. (2004). Temporal stability of the eating disorder examination questionnaire. International Journal of Eating Disorders.

[bib23] Riley C., Shafran R. (2005). Clinical perfectionism: A preliminary qualitative analysis. Behavioural and Cognitive Psychotherapy.

[bib24] Shafran R., Cooper Z., Fairburn C.G. (2002). Clinical perfectionism: A cognitive-behavioural analysis. Behaviour Research and Therapy.

[bib25] Shafran R., Cooper Z., Fairburn C.G. (2003). “Clinical perfectionism” is not “multidimensional perfectionism”: A reply to Hewitt, Flett, Besser, Sherry & McGee. Behaviour Research and Therapy.

[bib26] Vitousek K., Manke F. (1994). Personality variables and disorders in anorexia nervosa and bulimia nervosa. Journal of Abnormal Psychology.

